# A Monoiodotyrosine Challenge Test in a Parkinson’s Disease Model

**DOI:** 10.32607/actanaturae.11399

**Published:** 2021

**Authors:** A. R. Kim, E. N. Pavlova, V. E. Blokhin, V. V. Bogdanov, M. V. Ugrumov

**Affiliations:** Koltzov Institute of Developmental Biology of Russian Academy of Sciences, Moscow,119334 Russia

**Keywords:** Parkinson’s disease, early diagnosis, challenge test, monoiodotyrosine, MPTP

## Abstract

Early (preclinical) diagnosis of Parkinson’s disease (PD) is a major challenge in modern neuroscience. The objective of this study was to experimentally evaluate a diagnostic challenge test with monoiodotyrosine (MIT), an endogenous inhibitor of tyrosine hydroxylase. Striatal dopamine was shown to decrease by 34% 2 h after subcutaneous injection of 100 mg/kg MIT to intact mice, with the effect not being amplified by a further increase in the MIT dose. The selected MIT dose caused motor impairment in a neurotoxic mouse model of preclinical PD, but not in the controls. This was because MIT reduced striatal dopamine to the threshold of motor symptoms manifestation only in PD mice. Therefore, using the experimental mouse model of preclinical PD, we have shown that a MIT challenge test may be used to detect latent nigrostriatal dysfunction.

## INTRODUCTION

The pathogenesis of Parkinson’s disease (PD), a frequent neurodegenerative disorder, is based on the degradation of the brain’s dopaminergic nigrostriatal system that regulates motor function [1]. PD is characterized by a prolonged asymptomatic preclinical stage during which there is activation of the mechanisms that compensate the insufficiency of the nigrostriatal system [2]. Only 20–0 years after the disease onset, when the dopaminergic neuronal loss in the substantia nigra (SN) has reached more than 50%, and the striatal dopamine level has decreased below its threshold value (20–0% of the control level), does the patient develop specific motor symptoms that enable the diagnosis to be made [3].

In this context, of great importance is the development of a method for detecting latent neurodegeneration in the nigrostriatal system to diagnose PD long before the disease transits to its irreversible clinical stage. One of the most promising approaches is a challenge test involving short-term and reversible inhibition of tyrosine hydroxylase (TH), the key enzyme of dopamine synthesis [4]. The use of such an inhibitor at a dose that lowers the striatal dopamine level by 30–40% relative to its control values will not cause motor disorders in healthy people. In turn, the striatal dopamine level at the preclinical PD stage is initially reduced and its further decrease by an inhibitor will lead to hitting the threshold of motor symptoms manifestation, which enables the diagnosis to be made [5].

As a challenge agent, we used monoiodotyrosine (MIT), a reversible TH inhibitor that is present in the body as an intermediate in the synthesis of thyroid hormones [6]. Unlike synthetic inhibitors, such as α-methyl-*p*-tyrosine, MIT is of endogenous origin and undergoes rapid metabolism, which minimizes the duration of dopamine synthesis inhibition and reduces the risk of side effects [7].

The purpose of this study was to experimentally develop a MIT challenge test for the detection of latent neurodegeneration in a preclinical mouse model of PD. As a model, we used a neurotoxic preclinical PD model, developed earlier in our laboratory, based on dosed systemic administration of 1-methyl-4-phenyl- 1,2,3,6-tetrahydropyridine (MPTP), a precursor of the dopaminergic neuron neurotoxin [8], to mice.

## EXPERIMENTAL

We used 80 male C57BL/6 mice aged 2–2.5 months with a weight of 22–26 g (Stolbovaya nursery), which were kept under standard vivarium conditions with free access to food and water. Animal experiments were approved by the Ethics Committee of the Koltsov Institute of Developmental Biology of the Russian Academy of Sciences (Protocol No. 43 of November 19, 2020).

**Fig. 1:**
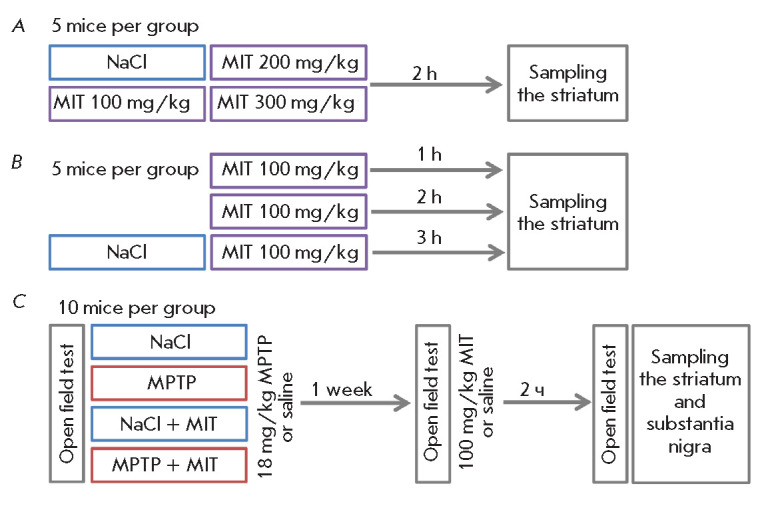
Experimental schemes: evaluation of the optimal dose of MIT (*A*) and time interval after its administration (*B*) to normal mice, and the MIT challenge test in the MPTP model of preclinical PD (*C*). MIT – monoiodotyrosine, NaCl – saline, MPTP – 1-methyl-4-phenyl-1,2,3,6-tetrahydropyridine

During the study, three experiments were performed (*Fig. 1*). In the first experiment, the effect of various MIT doses on the striatal dopamine level in intact mice was assessed and the optimal dose was selected for further analysis (*Fig. 1A*). In the second experiment, the selected MIT dose (100 mg/kg) was used to assess the pharmacodynamics and determine the optimal time interval for a maximum decrease in the striatal dopamine level in intact mice after administration (*Fig. 1B*). The third experiment was devoted to the development of a MIT challenge test in the neurotoxic MPTP mouse model of preclinical PD (*Fig. 1C*).

MIT (hereinafter, all reagents are from Sigma- Aldrich, USA) was dissolved in a physiological solution (0.9% NaCl) containing 5% ascorbic acid and 0.5% dimethyl sulfoxide and administered subcutaneously to animals at the indicated doses. The control groups received a similar solution without MIT. To simulate PD at the preclinical stage, mice were once subcutaneously injected with MPTP at a dose of 18 mg/kg [8]. The control groups received physiological saline.

The locomotor activity of the mice was assessed based on measures of the distance traveled and the number of rearings in an open-field behavior test. For adaptation, mice were transferred to a behavior testing room 2 h before the start of the test. The open-field test was performed using a PhenoMaster automated system (TSE Systems, Germany) for 6 min. The parameters were calculated using the supplied software.

To collect the nigrostriatal system structures, isoflurane anesthetized mice were decapitated and the dorsal striatum and SN were isolated from the brain according to the previously described procedure [8]. Samples of the brain structures were weighed, frozen in liquid nitrogen, and stored at –70°C. The dopamine concentration in the samples was measured by highperformance liquid chromatography with electrochemical detection according to [9].

Data are presented as a mean (a percentage of the control values) ± standard error of the mean. Data normality was examined using the Shapiro–Wilk test. Statistical analysis of the results was performed with the one-way ANOVA method, parametric Student’s* t*-test, or nonparametric Mann–hitney test using the GraphPad Prism 6.0 software package (GraphPad Software, USA). *P *≤0.05 was used as the statistical significance.

## RESULTS AND DISCUSSION

**Selection of the effective dose of MIT and the time after its administration**

**Fig. 2:**
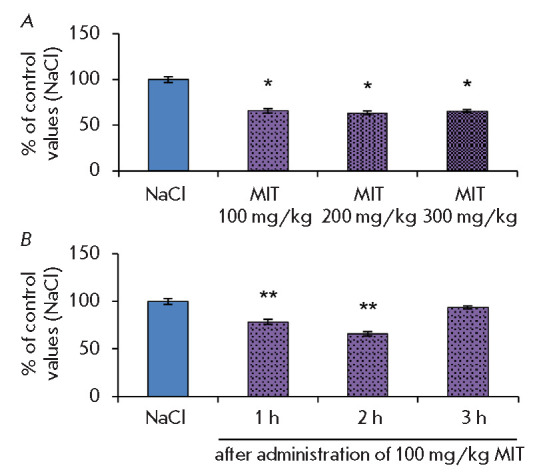
dopamine in mice 2 h after administration of various MIT doses (*A*) and 1, 2, or 3 h after administration of 100 mg/kg MIT (*B*). *p  < 0.05 compared to controls (NaCl); **p  < 0.05 compared to controls and all other groups. MIT – monoiodotyrosine

During selection of the MIT dose, 100 mg/kg MIT was found to provide the maximum decrease in the striatal dopamine concentration in normal mice (34% of the control level) (*Fig. 2A*). However, a further increase in the MIT dose, up to 200 and 300 mg/kg, did not lead to a further decrease in the dopamine level (*Fig. 2A*), which indicates TH saturation with the inhibitor and the absence of a linear MIT dose-dependency in this range. Therefore, 100 mg/kg was chosen as the effective MIT dose for further experiments.

An analysis of time intervals revealed that the striatal dopamine concentration decreased by 22% compared to that in the controls 1 h after MIT administration and by 35% after 2 h; after 3 h, the dopamine level was completely restored to its control values (*Fig. 2B*). These results confirm the short duration and reversibility of the MIT inhibitory effect on TH in the striatum. Therefore, a time interval of 2 h after inhibitor administration was chosen for the development of the MIT challenge test.

Interestingly, the same dose (100 mg/kg) of α-methyl-*p*-tyrosine was previously shown to reduce the striatal dopamine level more efficiently, by 40.2%, 4 h after its administration [5]. In this case, according to *in vitro *estimates, MIT is a more effective TH inhibitor in comparison with α-methyl-p-tyrosine [10]. Apparently, faster metabolism of MIT under *in vivo* conditions limits its inhibitory effect on TH.

**Challenge test in an experimental preclinical PD model**

An important factor in modeling PD is a precisely identified threshold of neurodegeneration at which motor symptoms appear. This is a loss of 50–60% of dopaminergic neuronal bodies in the SN and a decrease in the number of their axons and the striatal dopamine concentration by 70–80% compared to those in the controls [8]. Therefore, we chose an unchanged motor activity of animals in the open-field test, unchanged nigral dopamine content, and a decrease in the striatal dopamine level by less than 70% as the key parameters of a preclinical PD model.

**Fig. 3:**
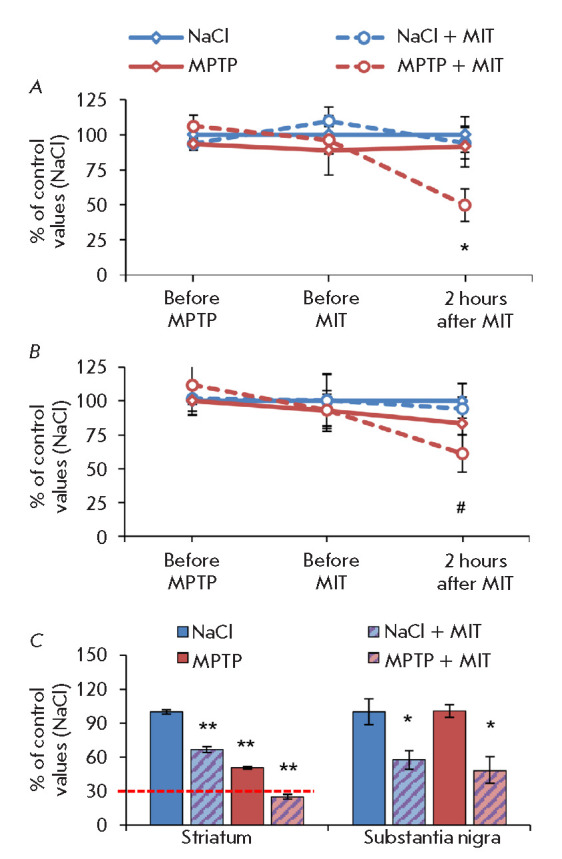
Total distance (*A*) and number of rearings (*B***) **in the open-field test and the dopamine level in the striatum and substantia nigra (*C*) of MPTP-treated or saline-treated (NaCl) mice 2 h after administration of 100 mg/kg MIT. *p  < 0.05 compared to controls (NaCl); **p < 0.05 compared to controls and all other groups; #*p* < 0.15 compared to controls

The traveled distance and the number of rearings in the open-field test in mice that received 18 mg/kg MPTP before MIT administration (i.e. 1 week after MPTP administration) did not differ from those in the controls (*Fig. 3 A,B*). In addition, administration of MPTP did not affect the dopamine level in the SN but decreased its level in the striatum by 49% (*Fig. 3C*), which is less than the indicated threshold of 70%. Thus, the key parameters of the experimental model corresponded to the characteristics of the preclinical PD stage.

Two hours after a subcutaneous injection of 100 mg/kg MIT, the mice modeling the preclinical PD stage developed motor symptoms: the distance traveled in the open-field test decreased by 50% relative to that in the control group (*Fig. 3A*), and the number of rearings reduced by 39% (*Fig. 3B*). In this case, there were no similar changes in the motor activity of either healthy mice receiving MIT or MPTP mice receiving physiological saline.

Apparently, this was because MIT caused a decrease in the dopamine concentration by 75% of the control level (i.e. below the threshold of motor symptom appearance) only in the striatum of mice in the preclinical PD model (*Fig. 3C*). Therefore, administration of MIT at the selected dose provoked motor symptoms in the preclinical PD model; i.e. in mice with latent insufficiency of the nigrostriatal system.

It is important to note that systemic TH inhibitors are relatively safe and have long been used in clinical practice. Another TH inhibitor, α-methyl-*p*-tyrosine, is used in the treatment of pheochromocytoma, a benign adrenal tumor [4, 5]. The drug doses used in this case lead to the inhibition of dopamine synthesis by 35–80%, and the duration of the daily intake varies from several weeks to several years [11], which indicates the absence of serious side effects even upon prolonged TH inhibition. However, there is evidence of potential neurotoxicity for MIT [12] and further research should pay particular attention to the analysis of the shortterm and long-term effects of its action on the brain and peripheral organs.

Therefore, the experimental preclinical mouse model of PD was a successful demonstration of the effectiveness of the MIT challenge test in the detection of a latent insufficiency in the dopaminergic nigrostriatal system. In this study, the optimal dose of MIT and the time after its administration were determined. The next stage in the development of a method for early PD diagnosis based on the MIT challenge test involves preclinical studies of pharmacokinetics, the toxicological properties, and long-term effects of MIT exposure.

## Abbreviations

DHBA: 3,4-dihydroxybenzylamine
HPLC-ED: high-performance liquid chromatography with electrochemical detection
MIT: monoiodotyrosine
MPTP: 1-methyl-4-phenyl-1,2,3,6-tetrahydropyridine
PD: Parkinson’s disease
TH: tyrosine hydroxylase
